# It's who you know: a review of peer networks and academic achievement in schools

**DOI:** 10.3389/fpsyg.2024.1444570

**Published:** 2025-02-24

**Authors:** Alison Black, Melissa F. Warstadt, Christoforos Mamas

**Affiliations:** Department of Education Studies, University of California, San Diego, La Jolla, CA, United States

**Keywords:** social network analysis, peer network effect, academic achievement, mixed methods, research review

## Abstract

Cultivating equitable academic achievement and enhancing academic functioning for youth is widely seen as one of the main missions of the public education system in the US, but educators may be overlooking critical factors for fully realizing this collective mission. This paper explores the emergent evidence on the impact of friendship and peer social networks on academic achievement and outcomes for students in K-12 public schools in the United States. In total, nine studies have been reviewed and presented. Findings suggest that peer social network selection and influence effects impact individual and group academic functioning and achievement outcomes across age groups in adolescence. Evidence across multiple studies suggests that structural effects and network position may be of special importance, particularly for low-achieving youth. However, due to publication bias issues and methodological limitations, findings should be taken cautiously. Social Network Analysis (SNA) is a quickly growing subfield of education research. There is a great need for more studies in the US, in particular those focusing on historically marginalized and underrepresented student populations (e.g., BIPOC and LGBTQ+ populations, highly mobile student groups, foster youth, students qualifying for special education support and accommodation, etc.). Another major gap identified is the need to focus on specific developmental stages, like elementary school or early adolescence, and varying environmental contexts.

## Introduction

Many educational, psychological, and social science theories have implied the critical role of relational and social capital in development and academic achievement. Certainly there are many factors influencing educational attainment (environmental, biological, psychological, etc.). Education research is often interdisciplinary. Different fields operate from different epistemologies and research traditions. While developmentalists may focus on psychological characteristics and psychometrics related to academic achievement, sociologists will likely be more interested in the sociocultural context of achievement. Social network theorists are preoccupied with relational elements and would argue these interactions supersede most, if not all, other factors for predicting outcomes like achievement. In the context of this review, we define academic achievement as referring to a student's performance and success in educational tasks, typically measured through grades, test scores, and completion of academic goals. It reflects how well a student has learned and applied knowledge in areas like reading, math, and other subjects. As noted above, we acknowledge that different perspectives emphasize various factors influencing achievement, such as psychological traits, sociocultural contexts, and the quality of social relationships.

Social Network Analysis (SNA) provides a set of tools that illuminate complex social processes, and works under the assumption that social capital can be mapped and quantified, visualizing related social processes that may be otherwise difficult to observe (Lin, 1999). By analyzing the connections between individuals within a network, SNA helps identify the patterns and strengths of relationships, such as who interacts with whom and how support or resources flow within a group. According to Social Network Theory (SNT), forms of capital (knowledge, attitudes, functioning, status, etc.) are distributed among relational ties within networks: the extent dependent on network composition and characteristics. For example, resilience can be fostered and distributed in communities of practice (Marin and Vazquez, 2012). An individual's location in a network can grant or deny access to such forms of social capital. Network position, structure, and composition can predict group and individual level outcomes (Borgatti et al., 2018).

In the past 15 years, social network scientists have developed innovative application methods for measuring and analyzing network characteristics and the many network construct variables influencing these social processes (i.e., selection, influence, network position, network composition, and other individual, school-level, and environmental factors). This review presents recent SNA findings on the relationship between peer social networks and academic achievement outcomes in public US K-12 schools. In this review, US K-12 schools refer to the public education system in the United States that serves students from kindergarten (K) through 12th grade (12). K-12 schools include elementary, middle, and high schools, and they provide foundational education in subjects like math, science, language arts, and social studies, preparing students for higher education or entry into the workforce.

## Methods

The current study observes research in the area of social network effects on academic performance in K-12 students. Much of the extant literature investigates other behavioral outcome variables, as opposed to academic outcomes. The current review was narrowed to focus on academic outcomes.

### Procedure

We began the literature search by systematically searching multiple databases in the fields of education and social sciences. We decided to set the search criteria to 2005–2020. To find relevant articles we used the following terms in various combinations: social network analysis, social network, high school, middle school, elementary school, K12, academic achievement, academic outcome, academic development. To exclude non-academic outcomes we utilized the “NOT” function and listed terms such as “risky behaviors” (e.g., smoking/tobacco use) that are often used in social network studies. From those articles, we used reference lists and “cited by” lists to find additional relevant articles.

We noted that many studies focused on high school students and measured academic outcomes without utilizing social network data and analysis methods. After reviewing the abstracts, we excluded literature not addressing students' social networks and academic outcomes. Articles linking social involvement to academic outcomes were included as they focused on social dimensions. Additionally, many studies were conducted outside the U.S., and only those referencing inclusive educational settings similar to U.S. standards were included. For the final review, studies had to meet these criteria: (1) conducted in a public, K-12, US setting, (2) included primary social network data through quantitative, qualitative, or mixed-methods approaches, (3) had an academic performance outcome variable, (4) utilized social network analysis techniques, and (5) were published in peer-reviewed journals between 2008 and 2020. These criteria resulted in nine articles for analysis, as shown in Table 1.

**Table 1:** Analysis of included studies.

**References**	**Association**	**Major findings**	**Key terms/ constructs**	**SNA approaches**	**Analysis tools**	**Setting/ context**	**Sample**
**Middle childhood**							
Cooc and Kim (2017)	Yes	Positive associations were strongest for children with lower initial reading skills	Social contagion, homophily	Longitudinal, individual and peer reading skills survey, peer nomination network survey	Descriptive analysis, regression models	Elementary, North Carolina	4,000+
**Early adolescence**							
Shin and Ryan (2014)	Yes	Influence effects were greater than selection effects	Selection, social influence, academic motivation, engagement and achievement.	Network structure: density, reciprocity, transitive ties	Longitudinal Stochastic actor-based model of social network analysis: SIENA R	6th graders, middle school	587
Ryabov (2011)	Yes	Peer social capital a significant predictor of achievement; “Segregated” peer networks may lead to higher achievement for some	Racial/ethnic segregation, peer social capital, educational attainment	Peer effect, peer social capital (average achievement of a peer network)	Multilevel modeling	National Sample, High schools and middle schools	19,117 Add Health
DeLay et al. (2016)	Yes	Peer influence improved writing and math performance in intervention settings	SEL intervention/ control, peer socialization, academic performance	Longitudinal, pre- and post-test	Stochastic actor-based model of social network analysis: SIENA R	Fifth graders, Elementary	631
**Middle adolescence**							
Bond et al. (2017)	Yes	Low-achieving networks associated with decreased achievement (no association for high-achieving)	Peer effects, selection, influence, homophily, shared environment	Longitudinal, interviews, friendship survey, reciprocity, ego centrality;	Regression, compare alter achievement in wave 1 to ego achievement in wave 2	Nationally sample of adolescent students grades 7–12	9,000+, add health
Maroulis and Gomez (2008)	Yes	Significant joint effect of network composition and network structure (not separately)	Lagged peer achievement, network composition, network structure (density/ triangulation), norm- enforcing & horizon expanding networks	Web-based social network survey; school records (GPAs, absences, standardized test scores), demographics	Regression models	High School, school within a school	439
Rambaran et al. (2017)	Yes	GPA and truancy explained by peer selection, peer influence, net of acceptance, connectedness; behaviors moderated by popularity and peer acceptance	Socialization, peer influence, behavior conformity, moderating effect of popularity (social standing)	Longitudinal, “Close Friends” Survey; popularity and acceptance scales	Longitudinal Stochastic actor-based model of social network analysis: SIENA R	High School–Southern California, semiurban, mostly white and Latinx	300+
Blansky et al. (2013)	Yes	Positive association: social contagion of academic success in social networks	Social contagion	Longitudinal, whole network computer-based survey, GPA (school data)	Regression analysis	Rural NY high school, mostly white	158
Duxbury and Haynie (2020)	Yes	Statistically significant and negative: school punishment and changes in peer networks explained behavior and lower achievement	Behavioral predispositions, punishment, labeling, and academic achievement, network mechanisms	Longitudinal, controlled for demographics, homophily and other network effects (outdegree, reciprocity, transitive triplets, and indegree popularity) to account for the influence of network structure on tie formation	Longitudinal network analysis and stochastic actor-oriented models (SAOM), Meta Regression	Two large high schools	1,909 add health

### Middle childhood (ages 6–12)

Researchers Cooc and Kim (2017) completed a longitudinal social network analysis study on secondary data to examine the relationship between peers' reading skills among 4,215 total second- and third-graders in 294 classrooms across 41 schools. The participants were drawn from a larger longitudinal study of an experimental reading program to reduce reading loss among low-income elementary students. Researchers analyzed the relationship between a measure of literacy skills [Dynamic Indicators of Basic Early Literacy Skills (DIBELS)] and a relational survey that documented whom children spoke to about reading. Children in the study were more likely to nominate peers with stronger reading skills, and positive associations between peer reading skills and individuals' reading achievement were strongest for those with lower initial reading achievement levels.

### Early adolescence (ages 10–15)

In a longitudinal study Shin and Ryan (2014) investigated selection and influence effects on academic motivation, engagement, and achievement. Researchers calculated GPA from report cards among 6th graders from fall to spring within one academic year. Findings from a network statistical analysis provided evidence that early adolescents in the sample sought friends (selection) who were similar to themselves in regard to academic achievement and self-efficacy. Researchers also found that friends became more similar to each other over time (influence) for all aspects of academic adjustment, except academic self-efficacy.

DeLay et al. (2016) controlled for peer selection in a longitudinal study investigating whether academic performance increased as a result of peer influence and social and emotional learning (SEL) intervention. This study took place in 6 schools within a district, in 5th grade classrooms. Participants included 631 fifth grade students from six elementary schools with 14 relationship building intervention (RBI) classrooms and eight control classrooms. Students were more likely to nominate friends and friends of friends in the RBI classrooms than control. Students in intervention classrooms became more similar to their friends' academic performance in writing. In math performance, lower performing students appeared to move toward their higher performing peers, with no evidence of the reverse. Higher performing students did not move toward their lower performing friends' scores.

In another longitudinal study, Ryabov (2011) examined social network effects among early adolescent racial and ethnic peer groups. Data were collected from the National Longitudinal Study of Adolescent Health (Add Health), collected at 3 different time points, from 80 high schools, 52 middle schools (feeder schools), for a total of 90,118 adolescents in the first data collection wave, and 20,745 in the second. The researcher used Hierarchical Linear Modeling (HLM) to predict academic achievement. Average achievement of peer networks was predictive of individual academic achievement for all students. In contrast with some earlier studies, homogeneous groupings for African American students may have led to improved academic achievement and attainment.

### Middle adolescence (ages 14–17)

In a longitudinal study with high schoolers, Blansky et al. (2013) collected data across two time points (January 2011 and 2012). On an electronic survey, participants (N=158 students in an eleventh grade class) self-reported GPA and nominated friends and acquaintances from a list. The surrounding social environment for each student was analyzed by categorizing academic progress, attendance, and disciplinary data, gathered from school records, across data collection points. The network study utilized visualizations of networks, network variables (density, transitivity, and cluster coefficient) and a linear regression analysis to model the relationship between social environment and academic outcomes. Friends' average GPA showed increased or decreased individual academic rankings over time, indicating social contagion effects.

Using a short-term longitudinal design, Rambaran et al. (2017) collected four waves of data over 2 years (fall and spring of ninth grade and tenth grade) at a semi-urban, moderately sized high school in southern California. Participants included 342 ninth-graders (174 boys, 168 girls, 50% European American, 35% Latino, 7% Asian or Pacific Islander). Researchers gathered truancy and GPA from school records. Stochastic actor-based modeling estimated selection effects based on students' individual characteristics and behaviors (i.e., GPA and truancy) while controlling for effects of network structure and homophily. Social acceptance moderated peer influence on academic achievement for reciprocal and one-way friendship ties; however, popular youth were influential on friends' truancy only in reciprocal friendships. In this study, participants tended to select friends with similar academic functioning, and became more similar over time, but selection contributed more than socialization to achievement similarity.

When looking at peer network effects on high school grades, researchers Maroulis and Gomez (2008) found a joint effect for both composition (the people in the network and their characteristics) and structure (density, triangulation). One of the high schools studied was a school-within-a-school model at a large urban high school, which showed to have more closed networks than typical large schools. Closed networks were shown to intensify peer effect in positive and negative directions, whereas density had a slight moderating effect. This study highlights the importance of analyzing joint effects in peer network research and the overall importance of school structure and students' connectedness for academic success.

In a large-scale study, researchers Bond et al. (2017) collected data from the National Longitudinal Study of Adolescent Health (Add Health), a representative national sample across 142 schools, grades 7–12. During Wave II interviews were conducted in students' homes to collect social networks, behaviors, and attitudes related to academic achievement. Regression models demonstrated a predictive relationship between academic achievement and centrality. In this study, embeddedness in a high-achieving network of friends was not associated with increased individual achievement, but embeddedness in low-achieving network predicted decreased individual achievement. Mutual friends displayed strongest associations. Based on their findings, the authors of this study argue that the drag effect is due in part to peer influence, not selection or environment alone.

Duxbury and Haynie (2020) investigated the social network mechanisms related to school punishment, labeling, and academic achievement in secondary schools. Situating their work in labeling theory and network theory they were hoping to shed light on social processes (peer influence dynamics) and mechanisms (network composition) linking labeling to behavioral change. Researchers collected data from the National Longitudinal Survey of Adolescent to Adult Health (AddHealth) collection between the years 1994–1996, with two waves of data collection, 1 year apart. The sample included 1,909 students in nine high schools with high survey response rates (over 75%) with much of their statistical power coming from two large schools. Networks were constructed for both waves. Researchers collected information on academic achievement (GPA) and behavioral predispositions (suspension in the past year; delinquency, depression, self-control, and impulsivity). They controlled for demographic characteristics (race, sex, age, and grade), homophily, and other network effects (outdegree, reciprocity, transitive triplets, and indegree popularity) to account for the influence of network structure on tie formation. They used a longitudinal network analysis, stochastic actor-oriented models (SAOM), and metaregression. Results showed significant negative associations: suspensions within 1 year were related to less tie formation with higher achieving peers compared to students who had not been suspended. Ties were more likely to form or sustain among students with similar levels of delinquency and achievement. Changes in students' friendship networks as a result of school suspension explained much of the main effect of school punishment on decreases in GPA.

### Social network mechanisms as predictors of academic achievement outcomes

All studies included in the mini review reported associations between peer networks and academic achievement. Some studies reported stronger associations over time, some reported a nuanced set of findings regarding the extent and direction of influence and selection effect on academic achievement. Peer social capital was a significant predictor of educational achievement (Ryabov, 2011). High peer reading scores were found to predict improved academic performance, especially for those with lower initial reading levels (Cooc and Kim, 2017). Some of the findings suggest social interventions can change peer networks in classrooms and, in turn, improve academic performance (DeLay et al., 2016).

In SEL intervention classrooms 5th graders nominated more friends and friends of friends than in non-intervention classes. They became more similar to friends' academic performance in writing. In math, lower performing students moved toward higher performing peers. Social network and SEL interventions resulted in changes in peer networks in classrooms and improved academic performance (DeLay et al., 2016). 6th graders sought friends (selection) with similar academic achievement and functioning; friends became more similar over time (influence) for all aspects of academic adjustment, except academic self-efficacy (Shin and Ryan, 2014). For high school and middle schoolers, average achievement of peer networks was predictive of achievement. Embeddedness in a low-achieving network was associated with decreased individual achievement (Bond et al., 2017). Predicting delinquency, ties were more likely to form and sustain among students with similar levels of delinquency, explaining changes in networks and lower achievement outcomes (Duxbury and Haynie, 2020). Friends' GPAs predicted individuals' GPAs in either direction (social contagion) (Blansky et al., 2013). Individuals tended to select friends with similar academic functioning and they became more similar over time (Rambaran et al., 2017).

#### Influence and selection effects

Several studies found patterns of peer influence or social contagion of academic success (Blansky et al., 2013; Bond et al., 2017; Maroulis and Gomez, 2008). Network density moderated the relationship between peer effect and academic performance (Maroulis and Gomez, 2008). Higher density and closed networks appeared to have intensifying effects. In one study, influence effects were greater than selection effects (Shin and Ryan, 2014), meaning that change over time as a result of exposure to peer social capital (behaviors, knowledge, etc.) rather than solely as a result of the propensity of students to select peers with similar academic achievement. Some studies found significant joint effects but not isolated effects for network composition and network structure on academic achievement (Maroulis and Gomez, 2008).

#### Unique outcomes for special populations

It is important to utilize the tools of social network analysis paired with mixed methods to explore student populations who have historically been excluded from the social benefits of institutions. For example, in contrast with some earlier studies, homogeneous groupings for African American students may have led to improved academic achievement and attainment (Ryabov, 2011). Qualitative, participatory youth studies about school-based social relationships and academic functioning/achievement may shed light on processes and explanations that network or quantitative measurements alone cannot describe. Racially, linguistically, and otherwise minoritized students, including students with disabilities, highly mobile youth, and those experiencing chronic absence and truancy, may have unique contextual factors to consider.

Students with less selection agency, especially those with lower academic achievement levels and those embedded in lower-achieving networks across age groups may be especially sensitive to peer influence and social network interventions (Cooc and Kim, 2017; DeLay et al., 2016; Duxbury and Haynie, 2020; Maroulis and Gomez, 2008; Rambaran et al., 2017). These studies also reiterate that school punishment and other institutional policies that constrain or impact peer selection can have a significant impact on academic achievement.

## Discussion

Across all reviewed studies, peer effect was found to be a determinant of individual academic outcomes, varying by extent and direction based on a number of dependent variables. There is likely no one dominant social capital-generating mechanism with regard to social networks and peer effect.

Data collection methods and sample size contributed to study results. Some of the studies found relatively small compositional or structural effects in terms of peer network influence in learning environments compared to what has been documented in the literature on descriptive studies. This may point to a need for more mixed methods, descriptive case studies as a middle ground between big data explorations with large internal subgroup compositional/structural variation and smaller descriptive studies that focus more on illustrating the social processes that explain composition and structure of networks that form.

Analysis methods may have also contributed to varied results in effect size and strength of association. Several studies employed newer longitudinal analysis, using Sienna in R (Snijders et al., 2010), which requires multiple data points or waves of observation. There are benefits and drawbacks to both large and small sample sizes. For example, larger sample size results in more plausible significance, in terms of peer effect on GPA, but may also include more variability in individual behavior and network composition and. Data at the school vs. classroom level yielded a predictable pattern: higher effects were found in smaller data sets and closed networks. Notwithstanding, because of variation in effect size dependent on contextual factors and variation of network characteristics, joint effects and moderating effects became of increased importance for interpreting findings for practical use, indicating that researchers might find more meaningful results by including analysis tools to measure joint or moderating effects.

Even with these nuances, the results of the present minireview make clear that relational aspects of school life (social networks) are determinants of academic achievement. The findings of the review also suggest benefits of interdisciplinary, mixed-methods, longitudinal approaches to measuring social processes related to academic achievement. This review also warrants a call for more studies replicating and expanding the application of social network analysis to large and small-scale studies of academic achievement and intervention, especially involving special populations and younger participants to learn more about social network behavior, cultural meaning, and youth perceptions.

Certain populations, especially those who have historically faced discrimination, exclusion, or additional social, economic, and educational barriers, may require unique research design methods for fully understanding the nuanced ways in which peer social networks impact academic outcomes. The same network studies and interventions that might work for a large portion of the US school population might not be effective for all students.

### Limitations

It is important to acknowledge the limitations of this mini review. First, there are limited studies that explore the specific relationship between peer networks and academic outcomes for K-12 students. This review highlights the importance of filling this gap in knowledge and including all stages of development in this field, especially with younger children. Due to the relatively limited number of studies in US K-12 schools utilizing SNA methods and measurements to investigate peer effects on academic achievement, it is difficult to generalize any specific social process patterns.

Additionally, from a methodological perspective, social network analysis relies heavily on quantitative methods and network structure indices. If we truly want to understand the impact of peer networks and friendships on children's and youth's school lives, researchers must include more robust qualitative and mixed methods studies. In doing so, we would be able to tell a more holistic story and better understand the cultural meaning behind school-based peer networks as a phenomenon (e.g., how youth conceive of their networks, choices, and schooling environments).

Though some studies included diverse samples, there does not yet appear to be a comprehensive field of studies representative of US demographic makeup in schools. The sample sizes varied from about 150–9,000. According to the search yields, research on young students' peer effect on academic achievement appears to be less available. There may also be a gap in the literature for early adolescence, a developmental stage characterized by shifts in social needs and behaviors. Traditional ways of studying subgroups, how we categorize them alone, reflects societal biases. Researchers should disaggregate demography in other ways that helps us tease out interactions among multiple factors (socio-economic status, geographic location, subgroups within larger racial/ethnic umbrella terms, intersectionality, other special populations and minoritized groups, etc.).

While peer networks appear to consistently predict academic achievement, it is likely an indirect effect. There may be more mediating factors that have not been explicitly measured in the selected studies. Potential factors may include selection, influence, and environmental opportunities and constraints (e.g., class roster, tracking, extracurriculars, school climate, identity-based experiences).

A common methodological limitation to SNA is that even longitudinal studies capture a series of snapshots in time at highly localized contexts that cannot be generalized. Improvements in longitudinal methods are helping to change this. Still, researchers might also consider applying various SNA methods in combination with others to studying positive school-based peer interventions, such as the effects of teen activism or leadership and group belonging and network effect on academic achievement.

## Conclusion

Peer interactions in schools matter, especially for low-achieving and historically excluded youth, yet we still know relatively little about the extent and direction of peer effect on academic achievement. This review highlights a need to replicate SNA studies across diverse contexts to continue assessing the varying role of social dynamics and environmental constraints in academic processes in US schools. More SNA/mixed-methods research is needed with younger students and understudied, marginalized populations who face unique social schooling contexts (students with disabilities, LGBTQ+, military-connected youth, foster youth, migrant youth, homeless youth, etc.) to discern how systemic-environmental factors impact historically excluded youth's access to selection and peer influence. Perhaps these constructs hold different meanings and functions for some youth.

Even so, an analysis of these present studies give substantive evidentiary support that even across developmental stages and individual characteristics, peer networks are important factors and determinants of academic success. Perhaps even more promising is the evidence that interventions can have a measurable impact on school-based social networks and subsequently influence academic performance. As a result, School leaders, counselors, and educators may want to consider and even leverage “relational health” (social networks) as important a factor in the education of their students as physical or mental health, and to consider the environmental, sociohistorical, and social-psychological factors that may enhance or limit relational connections (class schedules, attendance, disciplinary practices, school climate, classroom community practices) as a tool for improving academic outcomes for kids in schools.
